# Low vs. High Radioiodine Activity to Ablate the Thyroid after Thyroidectomy for Cancer: A Randomized Study

**DOI:** 10.1371/journal.pone.0001885

**Published:** 2008-04-02

**Authors:** Hanna O. Mäenpää, Jorma Heikkonen, Leila Vaalavirta, Mikko Tenhunen, Heikki Joensuu

**Affiliations:** Department of Oncology, Helsinki University Central Hospital, Helsinki, Finland; University of South Florida, United States of America

## Abstract

**Background:**

Radioactive iodine is commonly administered following thyroidectomy for differentiated thyroid carcinoma to ablate the thyroid remnant. The optimal administered activity of radioiodine is unknown.

**Methodology/Principal Findings:**

Adult subjects (*n* = 160) diagnosed with papillary or follicular thyroid carcinoma were randomly allocated to receive either 1100 MBq (30 mCi) or 3700 MBq (100 mCi) activity of radioiodine (^131^I) following thyroidectomy. The study participants were prepared for ablation using thyroid hormone withdrawal. Ablation was considered successful when serum thyroglobulin concentration was less than 1 ng/mL and no uptake was present in ^131^I scan. Ablation was successful following one administration of radioiodine in 42 (52%; 95% CI, 41% to 63%) of the 81 evaluable study participants who received 1100 MBq, and in 43 (56%, 45% to 67%) of the 77 subjects who received 3700 MBq activity (*P = *.61). There was no difference between the groups in the numbers of repeat radioiodine treatments needed to complete ablation (*P = *.27). The higher activity was associated with more nausea and taste disturbances, and a longer stay in a radioprotected isolation unit. None of the participants died from thyroid cancer during a median follow up of 51 months; three subjects in the 3700 MBq group and none in the 1100 MBq group were diagnosed with distant metastases during follow-up. In a meta-analysis of four randomized studies that compared the 1100 and 3700 MBq activities, the 1100 MBq activity tended to be associated with a higher risk of unsuccessful ablation (relative risk 1.148, 95% CI 0.974 to 1.353, *P* =  .10).

**Conclusions/Significance:**

The results provide no conclusive evidence that 3700 MBq activity is more effective for ablation of the thyroid remnant than 1100 MBq activity. The 3700 MBq activity is associated with more adverse effects.

**Trial Registration:**

ClinicalTrials.gov NCT00115895

## Introduction

Radioiodine ablation is recommended for most patients diagnosed with papillary or follicular thyroid carcinoma following thyroidectomy [1]. The potential advantages include eradication of occult carcinoma foci, and improved sensitivity of postoperative ^131^I scanning and serum thyroglobulin measurements for detection of recurrent disease [2]. Ablation is generally well tolerated, but may be associated with transient neck pain and oedema, sialadenitis, lethargy, impairment of gonadal functions, and rarely radiation-associated second cancer [2], [3]. Its efficacy in prevention of cancer recurrence has never been compared to surgery alone in a randomized trial, but some uncontrolled studies suggest that ablation may reduce substantially the risk of cancer recurrence [4].

The administered iodine activity needed to ablate the thyroid is controversial. The administered activities vary widely between centers from as small as 925 MBq (25 mCi) to as high as 7400 MBq (200 mCi) regardless of whether chosen empirically or based on dosimetry-guided techniques [2], [5]. Retrospective cohort studies [4] and randomized studies [6]–[10] suggest that low activities within the range of 1100 MBq (30 mCi) to 1850 MBq (50 mCi) might be as effective as higher activities, but the few randomized studies performed have been criticized for their small patient numbers and inadequate methodology [5], and the uncontrolled cohort studies may contain inherent biases. The relative benefits and harms of lower and higher administered activities thus remain unknown.

We evaluated the safety and efficacy of two commonly used radioiodine ablative activities (1100 MBq, 30 mCi; and 3700 MBq, 100 mCi) administered following thyroidectomy for differentiated thyroid cancer. We made an effort to accrue the majority of patients who underwent total or near total thyroidectomy in a single center. Since efficacy of ^131^I depends on the amount of the thyroid tissue left behind at surgery, we restricted the study within a patient population treated with thyroidectomy. To our knowledge, this is the first randomized study on thyroid ablation that addresses also adverse effects of radioiodine and duration of isolation required following administration.

## Methods

The protocol for this trial and supporting CONSORT checklist are available as supporting information; see Protocol S1 and Checklist S1.

### Study population

**Figure 1:**
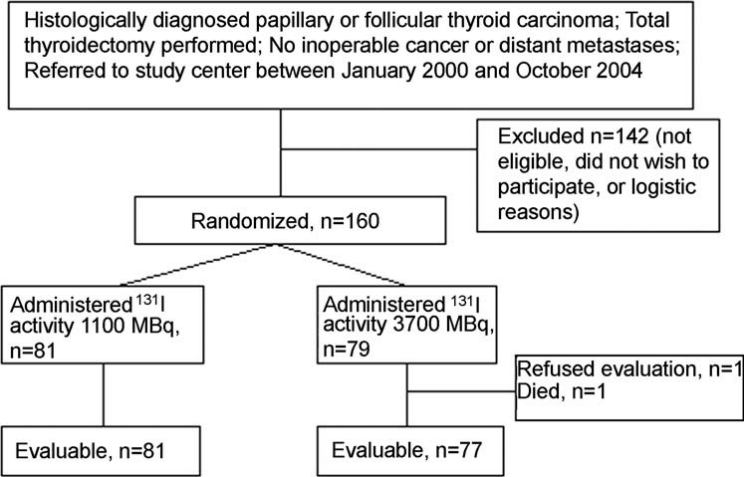
A consort diagram of the study.

One-hundred and sixty patients with histologically confirmed thyroid carcinoma were entered to this prospective, randomized, phase III, open-label, single-center study between January 2000 and October 2004. Patients were required to have undergone either total or near total thyroidectomy, have either papillary or follicular thyroid carcinoma diagnosed according to the World Health Organization (WHO) criteria [11], and they were expected to tolerate radioiodine administration. Patients who presented with macroscopic inoperable locoregional disease or with distant metastases were excluded, as well as pregnant women. The study participants are consecutive with the exception of the patients who were not eligible, those who selected not to participate, and a few patients who were not included because of logistic reasons (Figure 1). Staging work-up was performed four to five weeks after thyroidectomy and included physical examination, blood cell counts, blood biochemistry, serum thyroglobulin and triiodothyronine measurements, and usually chest X-ray. Postoperative neck ultrasonography or computed tomography were not mandated by the protocol.

All participants were randomly allocated to receive either 1110 or 3700 MBq activity of^ 131^I five to six weeks after thyroidectomy. At randomization, patients were stratified according to the presence or absence of histologically verified cervical lymph node metastases. Randomization was carried out centrally, and was done with a computer program where random digits were weighted based on the proportion of past randomizations to yield a balanced number of randomizations between the two groups in a concealed fashion.

The primary endpoint was successful thyroid ablation defined as 1) absence of abnormal uptake in a diagnostic whole body ^131^I scan, 2) undetectable (less than 1 ng/mL) serum thyroglobulin during both levothyroxine administration and thyroid stimulating hormone (TSH) stimulation, and 3) absence of palpable metastases in the neck. All three conditions had to be met for ablation to be considered as successful. We performed a second efficacy analysis (not specified by the study protocol) taking absence of abnormal activity in a diagnostic radioiodine scan as the sole criterion for successful ablation. Other endpoints in the study were tolerability of radioiodine treatment, the number of subsequent radioiodine treatments required to complete thyroid ablation, absorbed activity in the neck following ^131^I administration, and the time spent in an isolation unit following radioiodine administration.

All patients signed an informed consent prior to performing any of the study procedures. The study was approved by an ethics committee of Helsinki University Central Hospital, and conducted according to the guidelines of the Declaration of Helsinki. This trial was registered with an identifier NCT00115895 at www.clinicaltrials.gov.

### Study procedures

Thyroid stimulating hormone (TSH) stimulation was achieved by abstaining from levothyroxine administration for a minimum of four weeks prior to radioiodine administration. A diet containing low iodine content was recommended prior to ablation. The median serum TSH concentration was 69.0 mU/L (range, from 6 to 1180 mU/mL) when measured 0 to 14 days prior to ^131^I administration (in the 1100 MBq group the median was 67.5 mU/L, range, from 18 to 1180 mU/L; in the 3700 MBq group the median was 71 mU/L, range, from 6 to 242 mU/L; *P* = .55); 146 (91%) patients had TSH 30 mU/L or higher.

A small ^131^I activity (7.4 MBq, 0.2 mCi) was administered orally four to five weeks after thyroidectomy to measure radioiodine accumulation in the thyroid bed. The percentage uptake was measured with an Atomlab 950 Thyroid Uptake System (Biodex, New York, USA) 48 hours after administration of 7.4 MBq radioiodine. The ablative radioiodine activity (1100 MBq or 3700 MBq) was administered approximately five days later as oral capsules that were swallowed with water.

The patients stayed in a hospital isolation unit after radioiodine administration until the radiation protection limit required by the Finnish authorities was met; the remaining whole body ^131^I activity was required to be less than 400 MBq corresponding to approximately 15 µSv/h dose equivalent rate measured with a Geiger counter at one meter distance. Oral levothyroxine replacement therapy was initiated four days after radioiodine administration and adjusted individually based on serum TSH and free triiodothyronine measurements.

A whole-body iodine scan and single-photon emission computed tomography (SPECT) were performed four to seven days after ablative ^131^I administration using a double-headed Toshiba 7200 A/UI gamma camera (Toshiba Medical Systems, Nasu, Japan) equipped with high energy parallel-hole collimators. SPECT was done in 139 (87%) cases; it was not performed in 21 cases due to logistic reasons. The effective radioiodine activity half-life (T½)_eff_ in the neck was calculated using the physical (T½_phys_, eight days) and biological (T½_biol_) half-lives. The absorbed radiation dose to the thyroid bed was calculated in 121 (76%) cases [12].

The success of ablation was assessed four to eight months after ^131^I administration. Serum thyroglobulin levels were measured both while the patient was receiving levothyroxine and during TSH stimulation, which was achieved by abstaining from levothyroxine administration or by administering recombinant human TSH (rhTSH) for patients judged to tolerate abstaining from levothyroxine poorly (n = 11; four in the low activity arm and seven in the high activity arm). Serum thyroglobulin was analyzed using immunofluorometry (normal reference range, <24 ng/mL). In addition, a diagnostic whole body scan, carried out under TSH stimulation, was performed after administration of 185 MBq of ^131^I. It was not performed when serum thyroglobulin was detectable (≥1 ng/mL) while the patient was receiving levothyroxine.

Radioiodine ablation was repeated whenever serum thyroglobulin was detectable or when abnormal uptake was present in the thyroid bed or at extrathyroid sites in a diagnostic radioiodine scan. The repeat administered activity was the same as the first administered activity except for 15 patients in the 1100 MBq group who received 3700 MBq and for one patient in the 3700 MBq group who received 2960 MBq. When a second repeat treatment was required, all but two patients received at least 3700 MBq.

Adverse effects related to radioiodine administration, graded according to the National Cancer Institute Common Toxicity Criteria version 2.0, were recorded by the study participants on structured forms approximately four days before ^131^I administration, and four to seven days, two to three weeks, and three months following treatment.

### Statistical methods

We estimated that one radioiodine treatment would be successful in 40% of the study participants allocated to the 1100 MBq group and in 60% of those allocated to the 3700 MBq group. Using power (1-β) 80%, α .05 and a one-sided test, 160 participants were to be recruited to the study. An interim safety analysis was carried out when 80 patients had been evaluated for efficacy; a stopping rule was to be applied if a 50% difference in the success rate was observed in favor of the higher administered activity group.

Analyses were done according to the intention-to-treat principle with superiority approach. Frequency tables were analysed with the chi-square test or Fisher's exact test. The Bonferroni method was used to correct for multiple testing. Distributions of the days spent in an isolation unit were compared using the Mann-Whitney test. Confidence intervals for one proportion were calculated using normal approximation. A formal meta-analysis was conducted using pooled data from three previous studies and the present study. The combined relative risk (RR) and the 95% confidence interval for the RR were estimated with a fixed effect assumption. The heterogeneity between the studies was evaluated using Cochran's test. All *P* values are two-tailed. Statistical analyses carried out using an SAS® version 8.2 for Windows (SAS Institute Inc., Cary, NC, USA).

## Results

### Patients

Eighty-one patients received 1110 MBq of ^131^I and 79 received 3700 MBq as the ablative activity (Figure 1). Radioiodine was administered a median of 38 days after thyroidectomy (range, 27 to 124 days; 93% received treatment within six weeks from surgery).

All entered patients were eligible. Two patients in the 3700 MBq group could not be evaluated for completeness of thyroid ablation; one refused evaluation and another one died of acute myeloid leukaemia three months after radioiodine administration.

Randomization was balanced (Table 1). Three patients in the 1110 MBq group and one in the 3700 MBq group had no ^131^I uptake in the neck with undetectable serum thyroglobulin following thyroidectomy; none had ^131^I uptake comparable to an intact thyroid. Two patients allocated to the 3700 MBq group received only 2220 MBq due to a high radioiodine uptake in the thyroid bed after administration of a 7.4 MBq test dose (14.6% and 18.5% of the administered activity accumulated in the thyroid bed in these two cases).

**Table 1:** Patient and Tumor Characteristics

Characteristic		Low administered activity (1100 MBq, N = 81)	High administered activity (3700 MBq, N = 79)
		n (%)	n (%)
Age (yrs)			
	Median	49	45
	Range	23–79	18–90
Gender			
	Female	65 (80)	63 (80)
	Male	16 (20)	16 (20)
Tumor diameter (cm)			
	Median	1.9	1.5
	Range	0.2–6.0	0.2–7.0
	Not available	3	1
Histology			
	Papillary	74 (91)	72 (91)
	Follicular	4 ( 5)	7 ( 9)
	Both papillary and follicular	3 ( 4)	0 ( 0)
Cervical lymph node metastases			
	Present (pN0)	5 ( 6)	7 ( 9)
	Not present (pN+)	76 (94)	72 (91)
Multifocal cancer			
	Yes	25 (31)	29 (37)
	No	56 (69)	50 (63)
No. of lobes affected			
	One	64 (79)	59 (75)
	Both	17 (21)	20 (25)
Serum thyroglobulin prior to radioiodine treatment			
	<1 ng/mL	28 (35)	29 (37)
	≥1 ng/mL	53 (65)	47 (59)
	Not available	0 ( 0)	3 ( 4)
^131^I uptake in an isotope scan			
	No uptake	5 ( 6)	3 (4)
	Uptake	76 (94)	74 (94)
	Not available	0 ( 0)	2 ( 3)
Neck ^131^I uptake (%)			
	Median	1.8	2.3
	Range	0–20	0–19

### Treatment

Ablation was successful in 42 (52%; 95% CI, 41% to 63%) of the 81 evaluable patients who received 1100 MBq and in 43 (56%; 95% CI, 45% to 67%) of those who received 3700 MBq (*P* =  .61, Table 2).

**Table 2:** Success of Thyroid Ablation with Radioactive Iodine

Criteria	Proportion with successful ablation		*P*
	1110 MBq	3700 MBq	
	n/N^1^ (%)	n/N^1^ (%)	
Thyroglobulin <1 ng/mL, and Thyroglobulin <1 ng/mL, and no uptake in a diagnostic radioiodine scan^3^	42/81 (52)	43/77^2^ (56)	.61
No uptake in a diagnostic radioiodine scan^3^	49/77 (64)	55/72 (76)	.09

1 number of successful treatments (n) of the number of evaluable patients (N)

2 one patient selected not to be evaluated, one died of acute myeloid leukemia

3 diagnostic radioiodine scan was not performed in four cases in the 1100 MBq group and in five cases in the 3700 MBq group, since serum thyreoglobin was detectable while the patient received levothyroxine supplementation

When an exploratory analysis was carried out using absence of abnormal activity in a diagnostic radioiodine scan as the sole criterion of successful ablation, a slightly larger proportion of the patients treated with the higher administered activity tended to become successfully ablated (76%; 95% CI, 67% to 86% vs. 64%; 95% CI, 53% to 74%; *P*  = .09).

There was no significant difference in efficacy between the administered radioiodine activities in any of the post hoc subgroup analyses performed (male vs. female; age <45 vs. ≥45; papillary vs. follicular cancer; tumor diameter <4 cm vs. ≥4 cm; cervical nodal status negative, pN0 vs. positive, pN+; serum pretreatment thyroglobulin <10 ng/mL vs. ≥10 ng/mL; <20 ng/mL vs. ≥20 ng/mL; and neck ^131^I uptake <2% vs. ≥2 %). Of note, a single radioiodine treatment was unsuccessful in all 18 patients who had serum thyroglobulin 20 ng/mL or higher at baseline regardless of the activity administered, whereas it was successful in 83 (61%) of those patients who had a serum thyroglobulin concentration lower than 20 ng/mL (*P* <.0001). Similarly, using a cut-off value of 10 ng/mL for serum thyroglobulin, only six (19%) of the 32 first radioiodine ablation treatments were successful among the patients who had a serum thyroglobulin level 10 ng/mL or higher prior to radioiodine treatment as compared to 77 (63%) of those who had a lower level (*P* <.0001). Only two (17%) of the 12 patients who had cervical nodal metastases (pN+) had successful ablation following the first radioiodine administration as compared to 83 (57%) of the 146 patients who had no cervical metastases (pN0, *P*  = .029), and 7 (32%) of the 22 patients with a primary tumor diameter 4 cm or larger underwent successful ablation as compared to 76 (58%) of the 132 patients who had a smaller primary tumor at diagnosis (*P*  = .10).

### Repeat treatments

There was no difference between the groups in the numbers of repeat treatments needed to complete ablation. Thirty-eight (47%) patients allocated to the 1100 MBq group had one or more repeat treatments (27 had one, 11 had two or more repeat treatments) as compared to 32 (42%) patients allocated to the 3700 MBq group (22 had one, and ten had two or more repeat treatments; *P*  = .41). Three patients did not receive a repeat treatment despite incomplete ablation (1100 MBq group, one; 3700 MBq group, two).

### Adverse effects

Radioiodine was generally well tolerated, and the frequency of adverse effects decreased with time. All adverse effects were mild (grade 1 or 2) except for nausea, which was severe in four (6%) patients allocated to the 1100 MBq group and in seven (10%) in the 3700 MBq group (*P*  = .33). Patients who received a lower activity had less nausea and taste disturbances, and tended to have less pain in the salivary glands (Table 3). Ten patients (13%) in the 3700 MBq group and four (5%) in the 1100 MBq group required medication for neck pain (*P*  = .082).

**Table 3:** Recorded Common Adverse Effects Related to Radioiodine Treatment

Adverse effect		Radioiodine activity administered				*P*
		1110 MBq		3700 MBq		
		n/N^1^	(%)	n/N^1^	(%)	
Nausea^2^						
	5 days^3^	11/68	(17)	24/67	(37)	.0092
	2 weeks	5/63	( 8)	10/59	(18)	.13
	3 months	1/64	( 2)	2/57	( 4)	.60
Pain in the neck						
	5 days	21/68	(30)	26/67	(40)	.33
	2 weeks	18/63	(29)	16/59	(27)	.86
	3 months	7/64	(12)	10/57	(27)	.35
Pain in salivary glands						
	5 days	10/68	(15)	18/67	(26)	.08
	2 weeks	7/63	(12)	10/59	(16)	.35
	3 months	1/64	( 2)	2/57	( 4)	.60
Altered taste						
	5 days	12/68	(18)	10/67	(15)	.67
	2 weeks	9/63	(14)	17/59	(28)	.050
	3 months	0/64	( 0)	4/57	( 6)	.047
Altered smell						
	5 days	5/68	( 7)	6/67	( 9)	.73
	2 weeks	5/63	( 8)	3/59	( 5)	.72
	3 months	0/64	( 0)	0/57	( 2)	1.00
Dry eyes						
	5 days	5/68	( 7)	10/67	(15)	.16
	2 weeks	4/63	( 6)	7/59	(13)	.29
	3 months	5/64	( 8)	8/57	(15)	.27
Dry mouth						
	5 days	8/68	(13)	15/67	(21)	.10
	2 weeks	4/63	( 6)	4/59	( 7)	1.00
	3 months	4/64	( 6)	3/57	( 5)	1.00

1 number of patients with reported adverse effects (n) of the number of evaluable patients (N)

2 nausea was graded severe (gr. 3) in four and seven cases in the 1100 and 3700 MBq groups, respectively

3 time from radioiodine treatment administration to evaluation

### Duration of stay in an isolation unit

A higher administered activity was associated with a longer stay in an isolation unit (Table 4). The median duration of stay was two days (range, from two to four days) in the 1100 MBq administered activity group and three days (range, from two to six days) in the 3700 MBq group (*P* <.0001).

**Table 4:** Duration of Stay in an Isolation Unit

Number of days spent in an isolation unit	Radioiodine activity administered		*P*
	1110 MBq	3700 MBq	
	n (%)	n (%)	
2	47 (58)	2 ( 3)	<.0001
3	29 (36)	41 (52)	
4	5 ( 6)	32 (41)	
5	0 ( 0)	3 ( 4)	
6	0 ( 0)	1 ( 1)	

### Absorbed dose

We calculated a mean absorbed dose of 26 Gy (range, 1 to 224 Gy) in the thyroid bed in the 1110 MBq group and 76 Gy (range, 1 to 675 Gy) in the 3700 MBq group, but these figures are unlikely to be accurate, since the small size of the thyroid remnants together with the partial volume effect in scintigraphy prohibited reliable estimation of the volumes smaller than 3–4 cm^3^ in size, which was the case in the majority of cases. The measured mean absorbed dose per one MBq of administered ^131^I activity in the thyroid bed was similar between the allocation groups. We found no correlation between the success of ablation and the calculated absorbed dose.

### Cancer recurrence

The median follow-up time was 51 months from the date of randomization (range, from 18 to 77 months). Metastastic cervical lymph nodes were removed from 12 patients (the 1100 MBq group, n = 6; the 3700 MBq group, n = 6; *P = *.52). Sixty-eight (84%) patients allocated to receive 1100 MBq ^131^I were alive with undetectable serum thyroglobulin and with no abnormal uptake in a radioiodine scan at the end of follow-up as compared to 65 (82%) among those who received 3700 MBq. Three patients in the 3700 MBq group and none in the 1100 MBq group were diagnosed with distant metastases (*P* = .12, Fisher's exact test). None died from thyroid cancer during follow-up; three patients died of a competing cause (1100 MBq group, two; 3700 MBq group, one).

### Meta-analysis of published trials

**Figure 2:**
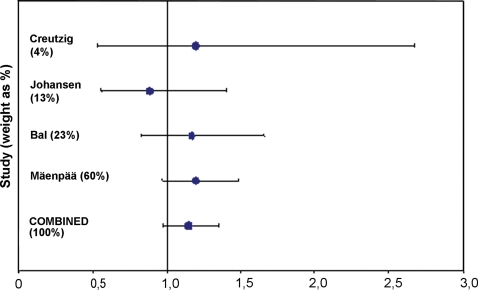
Meta-analysis of success of ablation in randomized prospective trials that have compared the 1100 MBq and 3700 MBq activities in ablation of the thyroid remnant. Relative risk (RR) with 95% confidence interval is shown. RR greater than 1.0 favors high activity treatment.

To the best of our knowledge, the studies listed in Table 5 are the only ones that have compared the 1100 MBq and 3700 MBq radioiodine activities in randomized prospective trials [13]. There was no significant heterogeneity between the four studies (*P* = .71, Cochran's test). The success rate for thyroid remnant ablation was 71.3% with the 3700 MBq activity and 62.1% with the 1100 MBq activity in a meta-analysis of the four studies. The relative risk (RR) for unsuccessful thyroid remnant ablation tended to be greater after administration of the smaller activity (RR 1.148, 95% confidence interval 0.974 to 1.353, *P* = .10; Figure 2).

**Table 5:** Reported Ablation Success Rates by Radioiodine Activity in Randomized Studies Comparing Low (1100 MBq) and High (3700 MBq) Administered Activity

Study	Proportion of patients successfully treated^1^ by activity	
	∼1100 MBq (30 mCi)	∼3700 MBq (100 mCi)
	% ( n/N^2^)	% (n/N^2^)
Creutzig et al. (1987)	50 (5/10)	60 (6/10)
Johansen et al. (1991)	58 (21/36)	52 (14/27)
Bal et al. (1996)	63 (17/27)	74 (28/38)
Present study	64 (49/77)	76 (55/72)
Total	61 (92/150)	70 (103/147)

1 assessed as absence of abnormal activity in radioiodine scan

2 number of successfully treated patients (n) of the number of evaluable patients (N)

## Discussion

We found no conclusive evidence that the higher radioiodine activity (3700 MBq) is more often associated with successful thyroid remnant ablation than the smaller activity (1100 MBq) when administered following thyroidectomy. In general, the smaller administered radioiodine activity was better tolerated. Six prior randomized studies have evaluated radioiodine activity in thyroid ablation [6]–[10], [14]. Five of these studies were small and included 20 to 138 patients each [6]–[9], [14]. The only large trial accrued 509 patients diagnosed with papillary or follicular thyroid cancer in Northern India [10]. In this study the participants were divided into eight groups treated with different activities ranging from 555 MBq (15 mCi) to 1850 MBq (50 mCi). A higher success rate (81.6%) was reported with activities of 925 MBq or greater as compared to lower activities (555 or 740 MBq, 61.8%). However, interpretation of this trial is difficult, because 28% of the patients underwent subtotal thyroidectomy or hemithyroidectomy, postoperative neck radioiodine uptake was generally high, the timing of ablation ranged between one month and nine years after surgery, a single primary end-point was not used, and 18% fewer patients than expected were allocated to the two groups that received the least activity.

Three prior randomized trials have compared the 1100 MBq activity with the 3700 MBq activity [6]–[8]. Although these studies have been criticized for their small size and methodology [5], the results are in line with the present findings (Table 5). The results of the meta-analysis performed by us found that the success rate in thyroid remnant ablation as evaluated by radioiodine scan tends to be higher with the 3700 MBq activity than with the 1100 MBq activity. The relative risk of unsuccessful ablation was 1.148 with the 1100 MBq activity, but the 95% confidence interval crossed 1.0 and the difference was thus not statistically significant. When the data of the meta-analysis (RR 1.148, success rates 71.3% vs. 62.1%) are used to design a study aiming to demonstrate superiority of the 3700 MBq radioiodine activity over the 1100 MBq activity using a two-sided significance level of 0.05 and power of 80%, altogether 822 subjects would need to be entered to the study. The number of study participants required may be even larger than this when successful ablation is defined by undetectable serum thyroglobulin concentration and a negative radioiodine scan. All studies performed so far may thus have been inadequately powered to be able to reliably demonstrate a difference in success of thyroid ablation between these two radioiodine activities.

Three randomized trials have compared 1100 MBq (30 mCi) activity with 1800 MBq (50 mCi) activity,^8,10,14^ and two randomized trials 1800 MBq activity with 3700 MBq (100 mCi) activity.^8,9^ Although some trials suggest that a high activity is more efficacious,^9,14^ the pooled risk ratios are not statistically significant and the results are therefore consistent with there being a superior effect associated with a high activity or no difference (reviewed in Hackshaw et al.^13^).

Patients with a unifocal carcinoma 1.0 cm in diameter or less, node-negative, and no extension beyond the thyroid capsule have low long-term recurrence rate (<2%) and may not benefit from adjuvant radioiodine, whereas radioiodine is likely beneficial when carcinoma persists in the neck or elsewhere. Selective use of radioiodine may be recommended for patients who have disease between these two extremes [15]. The American Thyroid Cancer Association Guidelines Taskforce recommends radioiodine ablation for stage III and IV disease (defined as American Joint Committee on Cancer 6^th^ edition), to all patients with stage II disease younger than age 45 years and most patients with stage II disease 45 years or older, and to selected patients with stage I disease [1].

The National Compehensive Cancer Network (NCCN) guidelines recommend use of 30 to 100 mCi radioiodine in cases of papillary, follicular or Hϋrthle cell carcinoma ≥1 cm in diameter, with nodal or distant metastases, or with aggressive histology when there is suspected or proven thyroid bed uptake in total body radioiodine scan after thyroidectomy [16].

Higher administered activity was associated with more nausea and taste disturbances. The absolute risk for radioiodine-induced cancers has not been well established, but the risk of any second primary cancer after initial diagnosis of thyroid cancer is increased approximately 30% over that of the general population [17], [18], and the risk appears to increase with increasing cumulative administered activity [18]. Radioiodine treatment may be associated with transient hypospermia and amenorrhea; permanent gonadal damage has been observed with cumulative activities exceeding 18.5–22.2 GBq [19], [20].

Only three patients developed distant metastases during the follow-up. This finding suggests that if the time to distant recurrence or overall survival are selected as the study primary endpoints, the study size needs to be very large, and it is doubtful whether such a study will be conducted. However, studies larger than the present one addressing radioiodine activity are warranted to exclude small but still clinically significant differences in efficacy between small and high administered radioiodine activities. The present study was a single-center trial, which is a limitation of the study, and further studies addressing radioiodine activity should preferentially be carried out in a multicenter setting. We used serum thyroglobulin levels as the basis of evaluation of success of radioiodine ablation, because whole body ^131^I scanning generally has low sensitivity after radioiodine ablation [15], [21], [22].

In conclusion, the results of the present trial and the meta-analysis provided no conclusive evidence that administration of 3700 MBq activity is more effective for ablation of the thyroid remnant than 1100 MBq activity among patients treated with total thyroidectomy for differentiated thyroid carcinoma. Administration of the smaller activity is associated with fewer common adverse effects and a shorter stay in a radiation protection unit, and does not result in more repeat treatments. The relative benefits and harms of using high (3700 MBq) as compared to low (1100 MBq) radioiodine activity remain inadequately studied, since none of the prior studies have addressed important aspects in clinical decision-making such as side effects of radioiodine administration, duration of isolation, or cancer recurrence rates after radioiodine administration.^6,7,8^ Evidence for selection of one or the other of the two radioiodine activities thus remains inconclusive. Importantly, the anti-cancer efficacy of different radioiodine activities has not been addressed in large, adequately powered controlled clinical trials. Such trials should receive a high priority.

## Supporting Information

Checklist S1 CONSORT checklist
(0.05 MB DOC)

Protocol S1 Trial Protocol
(0.07 MB DOC)
