# Supplementary material for: Low vs. High Radioiodine Activity to Ablate the Thyroid after Thyroidectomy for Cancer: A Randomized Study
Source: PLoS One. 2008 Apr 2;3(4):e1885. doi: 10.1371/journal.pone.0001885 (PMC2270902; doi:10.1371/journal.pone.0001885)
Supplement: Protocol S1 — Trial Protocol (0.07 MB DOC) [file pone.0001885.s002.doc]

**Study protocol**

**Effect of radioiodine activity in thyroid ablation following thyroidectomy for differentiated thyroid carcinoma**

Investigators:

Hanna Mäenpää, M.D.

Jorma Heikkonen, Ph.D.

Mikko Tenhunen, Ph.D.

Heikki Joensuu, M.D., professor

Contact information:

Hanna Mäenpää, M.D.

Department of Oncology,

Helsinki University Central Hospital

Haartmaninkatu 4

FIN00029 Helsinki, Finland

Phone: 09-4711, 0-050-4271838

hanna.maenpaa@hus.fi

**Background**

Some normal thyroid tissue is virtually always left behind in the neck following thyroidectomy for differentiated thyroid cancer. This thyroid tissue may or may not contain cancer. Lymph nodes of the neck may also contain metastatic cancer cells that have not been removed at surgery. Radioiodine is therefore usually administered postoperatively to eradicate both normal thyroid tissue and any remaining cancerous tissue.

The activities of radioiodine administered vary markedly in different countries ranging from 1.1 GBq to 7.4 GBq (from 30 to 200 mCi). Such substantial variation in radioiodine activity administered is due to a lack of properly conducted studies in this field. Only three randomized studies have been published, but these studies appear to have methodological shortcomings (Roos et al. 1998). In a small randomized study reported by Johansen et al. 36 patients were treated with radioiodine activity of 1,073 MBq (29 mCi) and 27 patients with 3,700 MBq (100 mCi). Ablation turned out to be equally effective in both groups (58% vs. 52%, respectively), but drawing reliable conclusions from this study is difficult. In another study (Creutzig et al. 1987) radioiodine activities of 1.1 GBq (30 mCi) and 3.7 MBq (100 mCi) were compared in a cohort of 20 patients who did not have metastatic lymph nodes in the neck. No difference in outcome was detected, but the size of the study was again very small. The largest randomized study has been carried out by Bal et al. (1996). In this study the randomization procedure may not have been appropriate, because the patients´ ability to pay for hospital costs influenced randomization, and similarly the radioiodine activity available at the hospital at the time of study entry probably also affected randomization. One hundred and forty-nine patients took part in this trial, and the administered radioiodine activities were 1,110 MBq (30 mCi), 1,850 MBq (50 mCi), 3,700 MBq (100 mCi) or 5,550 MBq (150 mCi). Thyroid ablation was successful in 63% if the cases treated with 1,110 MBq and in 77% of those who received administered activity of 5,550 MBq.

There are thus little data available from randomized studies regarding the optimal dose of administered radioiodine activity needed to ablate the thyroid following thyroidectomy, and the studies carried out thus far appear to have methodological problems. Based on uncontrolled (nonrandomized) studies, the activity required for ablation might depend on the amount of thyroid tissue that is left behind at surgery (Maxon et al. 1992, Malpani et al 1996). This issue, too, is still inadequately addressed in clinical studies.

Radioiodine ablation may be associated with adverse effects, and the frequency and severity of adverse events may be dependant of the activity administered. The most common adverse events include sialadenitis (33%), and temporary taste and smell alterations (27%). Alopecia may occur in up to 28% of the cases. Haematological toxicity may also occur, but severe hematological toxicity is rare. The risk of secondary malignancy appears to be small (de Vathaire et al. 1997). The legislation for radiation protection calls for use of the minimal effective activities in the management of patients.

In sum, the optimal administered activity of radioiodine following thyroidectomy to ablate the thyroid is not known, and further studies are warranted. Unfortunately, research on this issue has been sparse. The best way to obtain reliable information regarding the administered activity is to carry out a prospective randomized clinical trial, where the most commonly used administered radioiodine activities are compared.

**Aims of the study**

The aims of the study are the following:

1. To compare efficacy of two administered activities of radioactive iodine in terms of ablating the thyroid following thyroidectomy. The compared activities will be 1,110 MBq (30 mCi; used e.g. in the United States of America and in France) and 3,700 MBq (100 mCi, the activity usually administered e.g. in Finland).

2. To compare subjective and objective adverse effects between the two groups; and to compare costs related to these treatments (assessed based on the number of days spent in a hospital isolation unit).

3. To evaluate factors that might be predictive for successful ablation, such as the number of metastatic cervical lymph nodes, and the measured absorbed dose at the thyroid bed.

**Study participant inclusion and exclusion criteria**

**The study inclusion criteria are the following**:

1. A total or near total thyroidectomy has been carried out done for either papillary or follicular thyroid cancer.

2. The patient must have read and understood the written informed consent provided prior to study entry. The patient must have signed the informed consent for study participation.

3. The study participant is estimated to tolerate administration of the study treatment (radioactive iodine), and he/she is anticipated to be able to tolerate the stay in an isolation unit.

**The study exclusion criteria are the following:**

1. Pregnancy.

2. Any psychiatric or physical illness that is considered to prevent a stay in a hospital isolation unit for approximately 3 days.

3. Presence of macroscopic cancer in the regional lymph nodes or in the surgical bed that is left behind at surgery; or presence of overt metastases.

**Treatment allocation groups**

The study participants will be randomly allocated to receive either 1,110 MBq (30 mCi) or 3,700 MBq (100 mCi) of radioactive iodine following thyroidectomy.

**Randomisation**

Randomisation is done using a computer program located at the Department of Oncology, Helsinki University Central Hospital. At randomization, patient’s name, the social security number, and the cervical lymph node status (N status) are recorded.

The study participants will be stratified at randomization based on the presence or absence of metastatic cervical lymph nodes at surgery (N+ = macroscopic or microscopic metastases present in the cervical lymph nodes; N0 = no cervical metastases are present).

**Study procedures**

Eligibility to the study will be assessed three to four weeks after surgery by a physician. Concomitant diseases, symptoms and physical status will be assessed. The potential study participants will be informed about the study both orally and in writing. Random allocation between the radioiodine activities [1.1 GBq (30mCi) or 3.7 GBq (100mCi)] may occur after the patient has signed an informed consent.

**Radioiodine administration schedule (please see also institutional guidelines for radioiodine administration)**

**Study Day: Procedure:**

Day -5 (Wednesday) 4 to 5 weeks from surgery The following blood tests will be carried out: serum thyroglobulin, thyroid stimulating hormone, free tri- iodothyronine (T3); blood haemoglobin, white cell count, platelet count; serum Na, K, creatinine, aspartate amino transferase (AST). 7.4 MBq radioiodine is administered orally.

Day -4 (Thursday) Filling of baseline symptom/toxicity questionnaire. Neck radioiodine uptake measurement.

Day 0 (Monday) Radioiodine treatment (either 1,110 or 3,700 MBq, administered as a capsule orally); Geiger counts measured on the neck.

Day +1 and +2 (Tue/Wed) Body Geiger counts measured; discontinuation of isolation allowed when measured total body activity is <15 uSv.

Day +4 (Friday) SPECT and Geiger measurements; filling of adverse event questionnaire; whole body iodine scan

Day +11 (Friday) Neck CT.

Day +14 (Friday) A telephone call to the patient to inform about the results of the scans, filling of the adverse event questionnaire.

Day +90 Hospital visit; includes response evaluation by the physician, filling of the adverse event questionnaire and laboratory tests (serum thyroglobulin, thyroid stimulating hormone, free tri- iodothyronine (T3); blood haemoglobin, white cell count, platelet count; serum Na, K, creatinine, aspartate amino transferase).

**Note**: In case metastatic disease is diagnosed in the whole body iodine scan taken following administration of the ablative dose (this is rare), a supplementary activity of 70 mCi will be administered to the patients who were allocated to receive 1,100 mBq (30 mCi). Patients allocated to the higher activity group (3700 MBq, 100 mCi) will not receive a supplementary dose, but the patients will be evaluated, and repeat radioiodine treatment will be administered if clinically required at approximately 4 months after the date of administration of the first dose.

**Adverse event questionnaire**

A form evaluating adverse effects related to radioiodine treatment (such as neck pain, taste disturbances, and nausea) will be filled before radioiodine treatment, and approximately 5 days, 2 weeks, and 3 months after the date of radioiodine administration. The adverse events will be graded in accordance of the NCI-CTC grading.

**Measurement of absorbed activity in the neck**

The radiation activity absorbed in the neck is estimated using Geiger detector counts, the volume of the target in the neck that accumulates activity as assessed using SPECT imaging, and by calculating the percentage uptake of radioiodine in the neck as compared to the whole body uptake (i.e. neck vs. whole body uptake) using the formula developed by Maxon et al. (1975). The value of information obtained from SPECT as compared with that obtained from conventional whole body imaging will be evaluated when the study has been ongoing for approximately for one year. The activity distribution, including the activities at the medulla, will be estimated by comparing SPECT imaging results with the anatomical information provided by CT images of the neck.

**Treatment evaluation and study endpoints**

Radioiodine ablation will be evaluated from the following points of view: 1) success of ablation and how often radioiodine treatment needs to be repeated to complete ablation 2) change in the serum thyroglobulin levels 3) adverse events of treatment (as assessed with questionnaires and blood tests) 4) as times of disease-free survival and overall survival, and 5) duration of stay in a hospital isolation unit.

Radioiodine treatment is deemed unsuccessful and needs to be repeated whenever

1) abnormal uptake (compatible with presence of thyroid tissue or cancerous tissue) is present in a diagnostic whole body ^131^I scan carried out with 185 MBq (5 mCi) activity after a minimum of 4 months following radioiodine administration, and a after a minimum of 4-week interruption of thyroxin administration

2) detectable (1 ng/mL or higher) levels of thyroglobulin are present in the serum 4 months or later from the date of administration; includes serum thyroglobulin levels measured under thyroid stimulating hormone (TSH) stimulation (achieved by interruption of thyroxine administration for a minimum of 4 weeks).

3) or palpable neck metastases are detected.

**Repeat treatment**

Repeat radioiodine ablation will be given using the same administered activity as in the first attempt to ablate the thyroid. However, patients who are diagnosed with either regional metastases (includes patients who have pathologic uptake outside of the thyroid bed that is not compatible with salivary gland uptake) or distant metastases will be treated with administered activity of 4.4 MBq (120 mCi). If metastatic disease is not present but three or more radioiodine treatments are required to complete thyroid ablation, the administered activity needs to be at least 3.7 MBq (100 mCi) when the third (or subsequent) attempts to ablate the thyroid are made.

**Study power**

We estimate that one radioiodine treatment is successful in 40% of the patients allocated to receive the smaller administered dose (1,100 MBq) and in 60% of the patients allocated to the higher activity (3,700 MBq) group. Using a power (1-β) 80%, α 0.05 and a one-sided log-rank test, 80 patients need to be recruited to each one of the study arms.

Estimation of patient accrual rate: The number of patients diagnosed with either papillary or follicular thyroid cancer at the Department of Oncology, Helsinki University Central Hospital, and treated with radioiodine after thyroidectomy ranges from 50 to 60 per annum. We estimate that 40 to 50 of these patients may fulfil the inclusion criteria of the study. The duration of the study accrual is estimated to be 3 to 4 years provided that a total of 160 patients are entered to the study.

An interim safety analysis: The study will be discontinued if a difference of 50% or greater will be detected in the proportions of patients whose thyroid is successfully ablated (with no need for retreatment) when one half or the study participants (80 patients) have been treated in the study (p is <0.01).

**References**

1. Alexander C, Bader JB, Schaefer A, et al. Intermediate and long.term side effects of high dose radioiodine therapy for thyroid carcinoma. J Nucl Med 39, 1551-1554, 1998

2. Bal C, Padhy AK, Jana S, et al: Prospective randomized clinical trial to evaluate the optimal dose of ^131^I for remnant ablation in patients with differentiated thyroid carcinoma. Cancer 77: 2574-2580, 1996

3. Creutzig H: High or low dose radioiodine ablation of thyroid remnants? Eur J Nucl Med 12: 500-512, 1987

4. Johansen K, Woodhouse NJ, Odugbesan O: Comparison of 1073 MBq and 3700 MBq iodine-131 in postoperative ablation of residual thyroid tissue in patients with differentiated thyroid cancer. J Nucl Med 32: 252-254, 1991

5. Roos DE, Smith JG: Randomized trials on radioactive iodine ablation of thyroid remnants for thyroid carcinoma – a critique. Int J Radiat Oncol Biol Phys 44: 493-495, 1999
